# A weakly supervised method for surgical scene components detection with visual foundation model

**DOI:** 10.1371/journal.pone.0322751

**Published:** 2025-05-27

**Authors:** Xiaoyan Zhang, Jingyi Feng, Qian Zhang, Liming Wu, Yichen Zhu, Ziyu Zhou, Jiquan Liu, Huilong Duan

**Affiliations:** 1 Key Laboratory for Biomedical Engineering of Ministry of Education, College of Biomedical Engineering and Instrument Science, Zhejiang University, Hangzhou, China; 2 The First Affiliated Hospital, Zhejiang University School of Medicine, Hangzhou, China; South China University of Technology, CHINA

## Abstract

**Purpose:** Detection of crucial components is a fundamental problem in surgical scene understanding. Limited by the huge cost of spatial annotation, current studies mainly focus on the recognition of three surgical elements ⟨instrument, verb, target⟩, while the detection of surgical components ⟨instrument, target⟩ remains highly challenging. Some efforts have been made to detect surgical components, yet their limitations include: (1) Detection performance highly depends on the amount of manual spatial annotations; (2) No previous study has investigated the detection of targets.

**Methods:** We introduce a weakly supervised method for detecting key components by novelly combining the surgical triplet recognition model and the foundation model of Segment Anything Model (SAM). First, by setting appropriate prompts, we used SAM to generate candidate regions for surgical components. Then, we preliminarily localize components by extracting positive activation areas in class activation maps from the recognition model. However, using instrument’s class activation as a position attention guide for target recognition leads to positional deviations in the target’s resulting positive activation. To tackle this issue, we propose RDV-AGC by introducing an Attention Guide Correction (AGC) module. This module adjusts the attention guidance for target according to the instrument’s forward direction. Finally, we match the initial localization of instruments and targets with the candidate areas generated by SAM, achieving precise detection of components in the surgical scene.

**Results:** Through ablation studies and comparisons to similar works, our method has achieved remarkable performance without requiring any spatial annotations.

**Conclusion:** This study introduced a novel weakly supervised method for detecting surgical components by integrating the surgical triplet recognition model with visual foundation model.

## Introduction

Surgical scene activities understanding in endoscopic surgery is a crucial issue in surgical data analysis. AI-based methods have facilitated the automatic identification of actions during surgery, providing assistance for surgeons in decision-making, planning and skill-teaching [1–3]. The surgical action triplet [4], defined as ⟨instrument, verb, target⟩, is a new paradigm for understanding surgical scene activities in a fine-grained modeling way. A considerable number of research [5,6] focus on predicting the presence of surgical triplets. Nonetheless, few studies investigate the spatial localization of surgical scene components ⟨instrument, target⟩ in surgical video frames.

In the past few years, several work [7], [8,9] have made efforts to detect components in surgical scenes. However, these methods share common limitations: (1) The performance of surgical components localization is significantly influenced by the amount of spatial annotations used for training. The outcomes from Endoscopic Vision Challenge [8] show that fully supervised methods, dependent on extensive spatial annotations, significantly outperform the weakly supervised methods lacking such annotations. Results in [9] also indicate a positive correlation with the amount of spatial annotations (The 60.2% increase in the amount of annotations results in the 13.2% improvement in performance). (2) The localization of targets is highly challenging due to their ambiguous boundaries and low discriminative features, leading to a focus solely on the localization of instruments, while ignoring the targets. Following these observations, we consider two research questions: (1) In addition to detecting instruments in surgical scenes, how can we achieve precise localization of the targets they acted upon? (2) Considering the high cost of manually annotating positions of instruments and targets, how can we achieve excellent localization performance without relying on spatial annotations?

**Fig 1:**
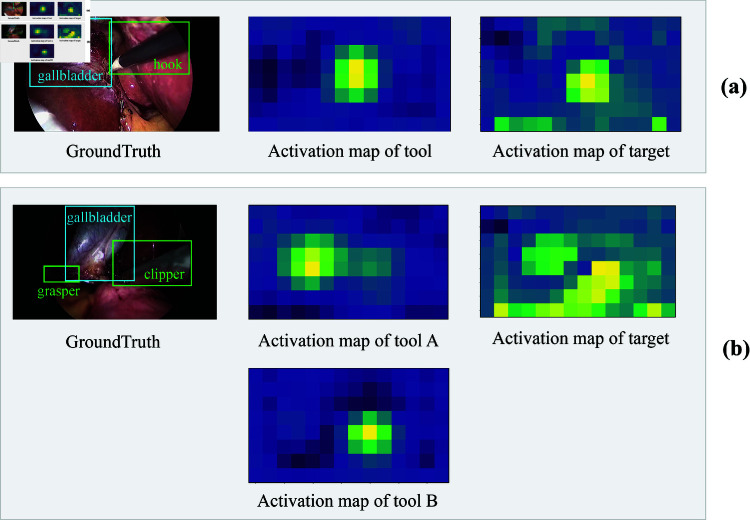
Instances of limitations in CAM-based target localization in RDV-det: (a) The positive activation area of the target is very close to the instrument; (b) Due to the considerable distance between two instruments, the positive activation area of the target exhibits an inability to “focus”.

The Segment Anything Model (SAM) [10], a pioneering foundational model for promptable segmentation, has recently attracted significant attention. Many recent studies [11–13] utilize SAM for downstream medical tasks, using its zero-shot learning capabilities to improve the training efficiency of medical vision models. The emergence of such visual foundation models [14,15] offers the potential to detect surgical components without spatial annotation. To address the research questions proposed above, we introduce a novel weakly supervised method that combines a surgical triplet recognition model and SAM. First, by setting appropriate prompts, we construct SAM’s Automatic Mask Generation pipeline to generate candidate regions for surgical components. Then, similar to the detection version of the surgical triplet recognition model Rendezvous (RDV), RDV-det [4], we preliminarily locate instruments and targets based on the positive activation areas in the Class Activation Map (CAM) [16]. However, due to the using of instrument’s class activation as position attention guide for target in Rendezvous [4], the positive activation area of target’s resulting CAM may converge towards the instrument, as shown in Fig 1 (a). This issue is more intractable when multiple instruments operate on the same target, different instruments’ CAMs may mislead the attention of target, resulting in the target’s positive activation failing to "focus" on the correct area, as shown in Fig 1 (b). To tackle this problem, we propose an Attention Guide Correction (AGC) module. Through shifting the original attention guide for the target, which is the instrument’s CAM, along the forward direction of the instrument, this module aligns the target’s resulting positive activation with its actual location, thus achieving more accurate preliminary localization. Finally, based on Hungarian algorithm [17] and designing appropriate matching costs, we match the preliminary localization of instruments and targets with the candidate regions generated by SAM, thereby facilitating precise detection of surgical scene components without the need of spatial annotations. To evaluate our method, we annotate the spatial bounds of instruments and targets on a subset of CholecT50 [18]. These annotations are only utilized for model evaluation and will be publicly available with this paper. Finally, ablation studies validate the effectiveness of the proposed modules, and comparisons with similar works demonstrate that our method achieves superior detection of surgical components without relying on spatial annotations. To summarize, we make the following contributions in this paper:

1. A novel weakly supervised method for detecting surgical components by combining the surgical triplet recognition model and the foundation model of Segment Anything Model (SAM).
2. We present the first exploration of locating the target acted upon by the instrument. The proposed Attention Guide Correction (AGC) module significantly enhances target localization performance through the automatic correction of position attention guides.
3. We match candidate regions from SAM without class labels to class activation maps. We utilize the strong generalization capabilities of the foundation model, achieving more accurate weakly supervised localization than CAM-based methods.
4. The proposed method eliminates the need for spatial annotations during training, thereby facilitating its extension to other laparoscopic surgery scenes.
5. Our approach achieves state-of-the-art results among all publicly available weakly supervised methods for detecting instruments. Moreover, its performance is comparable to those fully supervised methods trained on extensive spatial annotations.

## Related work

### Surgical action triplet recognition

The concept of the surgical action triplet originates from the existing surgical ontology, where each triplet is described as a combination of the used instrument, a verb representing the action performed, and the anatomy acted upon [19]. Surgical action triplets was introduced to surgical workflow analysis for improving surgical phase recognition by Katic *et al*. [20]. Nwoye *et al*. developed a deep learning model, Tripnet, for the automatic recognition of action triplets in surgical video frames [21]. This model introduced a mechanism called the Class Activation Guide (CAG), which uses the class activation of the instrument as appearance cues to guide the recognition of verbs and targets. Additionally, it projects the components of the triplet into a 3D interaction space (3Dis) to learn their association, thus achieving more accurate results. In this work, Nwoye *et al*. also introduced CholecT40, an endoscopic video dataset annotated with action triplets.

In a recent work, Nwoye *et al*. improved upon Tripnet [4]. The proposed model, Rendezvous (RDV), leverages the attention mechanism at two different levels to enhance the recognition performance of triplets. Firstly, it integrates the spatial attention mechanism with the Class Activation Guide module from Tripnet, introducing a new form of spatial attention called the Class Activation Guided Attention Mechanism (CAGAM). It focuses on using the instrument’s resulting class activation as a position attention guide for recognizing verbs and targets. To model the final triplet association, the RDV model adds a new form of semantic attention called Multi-Head of Mixed Attention (MHMA). This technique employs several cross and self-attentions to effectively capture the relationships between the three components within the surgical action triplet.

### Instrument detection

Utilizing a region-based convolutional neural networks, Jin *et al*. investigated the localization of instruments in laparoscopic surgery videos for the first time in [7]. They also introduced a new dataset, m2cai16-tool-locations, which extended the m2cai16-tool dataset with spatial bounds of instruments. An endoscopic vision challenge named CholecTriplet2022 [8] introduced a detection task, which required the localization of instruments in video frames and their accurate association with triplets. Since the challenge lacks instrument spatial annotations, the majority of the submitted methods utilize weak supervision to learn the instrument locations by exploiting the model’s class activation map (CAM), a few other methods take advantage of external datasets that provide complementary spatial annotations for instruments. The results indicate that compared to fully supervised methods, the performance of weakly supervised methods for instrument detection is unsatisfactory due to the imprecision of CAM-based localization. Among these, the best weakly supervised method achieves only 11.0% mean average precision (mAP), while the mAP of the top fully supervised approach is 41.9%.

A recently work [9] proposed a two-stage mixed supervised learning strategy for instrument localization and triplet association. This approach begins by learning target embeddings that fuse instrument spatial semantics and image features, then builds associations between detected instrument instances and target embeddings based on interaction graphs. Compared to the top methods in CholecTriplet2022 [8], this method achieves higher instrument detection accuracy with fewer bounding box instances. However, its detection performance is also highly dependent on the amount of spatial annotations used for training. To achieve an mAP of over 50% in instrument detection, more than 15,000 spatial annotated frames are used for model training.

### Adaptation of SAM for medical images analysis

A recently work introduced a foundation model called SAM for the task of promptable segmentation [10]. Leveraging its powerful zero-shot learning ability, SAM can segment images by inputting prompts in the forms of points, bounding boxes and masks. How-ever, directly applying SAM to medical image does not produce good results [22,23]. This is because the training data of SAM primarily consists of natural images. Some studies investigated how to adapt the SAM model for downstream medical image tasks. In-stead of fine-tuning the SAM model, Wu *et al*. proposed the Medical SAM Adapter (Med-SA) [12], which incorporates domain-specific medical knowledge into the segmentation model using a light yet effective adaptation technique. Li *et al*. proposed a fine-tuned SAM model for polyp segmentation named Poly-SAM [24]. They fine-tune SAM model on a collection of multi-center colonoscopy images. The fine-tuning strategy is to freeze SAM’s encoder and fine-tune only mask decoder.

In the field of laparoscopic surgery, Wang *et al*. examined SAM’s robustness and zero-shot generalizability in robotic laparoscopic surgery [23]. The evaluation results reveal that although SAM shows remarkable zero-shot learning ability with bounding box prompts, it struggles to segment the instrument with point prompts and unprompted settings. Another work introduced AdaptiveSAM for adaptive modifying SAM [13]. The proposed approach for fine-tuning AdaptiveSAM called bias-tuning requires a significantly smaller number of trainable parameters than SAM (less than 2%). Currently, the application of SAM in laparoscopic surgery typically requires experts to offer prompt-based inputs.

## Materials and methods

**Fig 2:**
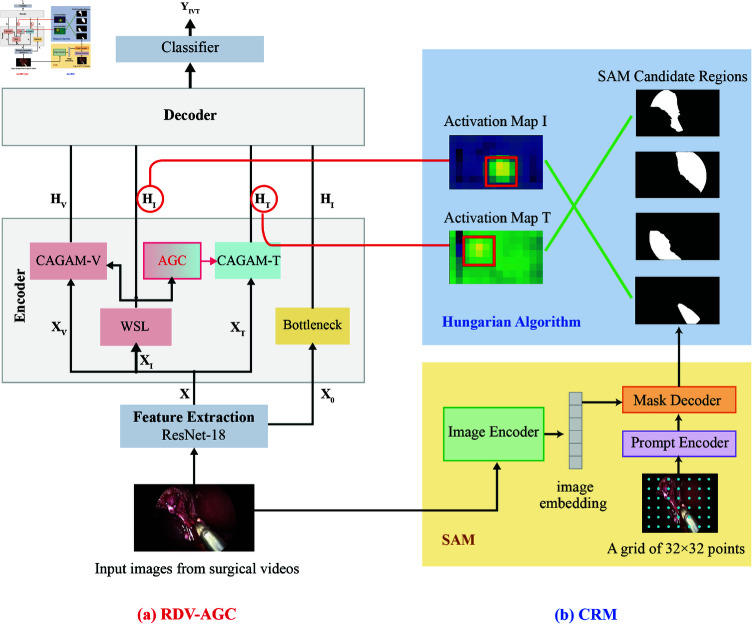
Architecture of our method: (a) RDV-AGC for surgical triplet recognition and preliminary localization; (b) Candidate regions generation pipeline and CRM mechanism.

**Fig 3:**
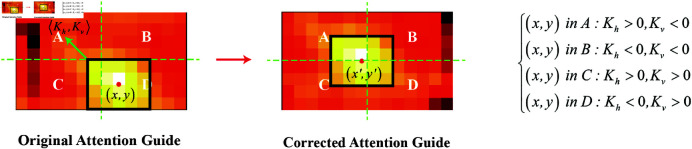
A detailed illustrator of the AGC module: For example, when the center of the instrument’s positive activation locates in the bottom-right area, we hypothesize that the operated target is most likely located at the upper left of the instrument, hence *K*_*h*_<0, $K_{v}<0$.

We design a weakly supervised deep learning model for the detection of crucial surgical components, instrument and target, as shown in Fig 2 and Fig 3. Our method is implemented through the following steps.

### SAM candidate regions generation

For each video frame, we use a modified version of SAM’s automatic mask generation pipeline [10] to generate candidate regions. First, we prompt SAM to generate all regions with a *N* × *N* regular grid of foreground points. Second, we filter the regions by predicted Intersection over Union (IoU) and stability, and remove redundant areas using Non-Maxima Suppression (NMS). We also observe that the masks from SAM contain minor, spurious components caused by reflections or water stains. To ignore these areas of no interest, we remove regions smaller than 2000 pixels. Finally, the remaining regions are ranked based on the average of their confidence and stability scores, and truncated to the top-10 region proposals. Ultimately, each frame will yield a collection of candidate regions, denoted as *Reg*_*s*_.

### Target class activation correction

The instruments’ class activation is utilized as a position attention guide for targets in RDV [4], inevitably causing that the positive activation area in target’s CAMs converge towards the instrument, as shown in Fig 1 (a). To make the positive activation of target align with the true position, we develop a recognition model named **RDV-AGC** by introducing an **A**ttention **G**uide **C**orrection ( **AGC**) module, as illustrated in Fig 2 and Fig 3. It channel-wisely translates the original attention guide (instrument’s CAM) in the forward direction of instrument by $\langle K_{h}$, $K_{v}\rangle$. The absolute values of *K*_*h*_ and $K_{v}$ represent the distance of shift in the horizontal and vertical directions, while the sign indicating the direction (positive for right/upward, negative for left/downward). The forward direction is inferred from instrument’s current position since they typically move from outside to inside the scene in laparoscopic surgery. For example, when the center of the instrument’s positive activation locates in the upper-right area, we hypothesize that the instrument will move towards the bottom-left. Therefore, the operated target is most likely located at the lower left of the instrument, hence *K*_*h*_<0, $K_{v}<0$. In scenes where multiple positive activation areas exist in the same CAM, the forward direction is determined by the area with the maximal average activation. For each activation point (x,y) in the original attention guide, the new coordinate $\left(x^{\prime },y^{\prime }\right)$ is given as follows:

$$\left\{ \begin{array}{c} x^{\prime }=\left(x+K_{h}\right)\mathrm{mod}W\\ y^{\prime }=\left(y+K_{v}\right)\mathrm{mod}H \end{array}\right.$$ (1)

Where *W* and *H* respectively represent the number of activation points in the horizontal and vertical directions in CAM. Our model RDV-AGC is trained end-to-end. We adopt a training strategy similar to "fine-tuning" to accelerate the model’s convergence. Specifically, we initialize the Feature Extractor module and Weakly Supervised Localization (WSL) module [4] with corresponding parameters from RDV which is pre-trained on the same dataset. The loss function follows the definition in RDV as Eq 2:

$$ℒ=L_{comp}+\rho L_{assoc}+\lambda L_{2}$$ (2)

Where *L*_*comp*_ represents the multi-task loss for multi-label classification of each triplet component, the detail of *L*_*comp*_ is provided in S1 Appendix. *L*_*assoc*_ represents the triplet association loss, which is modeled as a sigmoid cross-entropy. ρ is a warm-up parameter, and $\lambda L_{2}$ is the *L*_2_ normalized loss with a regularization weight decay.

### Preliminary localization

Utilizing CAM to identify objects’ discriminative regions is a commonly used method for weakly supervised localization [25–27]. The pipeline of preliminary locating surgical components from CAM is outlined as follows:

1. Extracting the CAMs from global max pooling (GMP) layer in both instrument and target branches.
2. Identifying all positive and locally maximal activation points from each CAM.
3. Constructing bounding boxes centered on these maximal activation points to enclose the surrounding positive activation areas.
4. Removing the redundant boxes by NMS.

We ultimately generate a collection of positive activation areas of instruments and targets in each frame, denoted as *Box*_*r*_.

### Precise localization

We match preliminary localization result $box\in Box_{r}$ with SAM candidate region $reg\in Reg_{s}$ by a mechanism denoted as **C**andidate **R**egion **M**atching ( **CRM**). Guided by the criterion of minimum matching cost, we make the matching utilizing Hungarian algorithm [17]. The matching cost is determined by the overlapping area of $box\in Box_{r}$ with $reg\in Reg_{s}$ and the average activation value of $box\in Box_{r}$. *C*_*i*,*j*_, one of the elements in cost matrix, its reciprocal is defined as:

$${C_{i,j}}^{-1}=Overlap\left(box_{i},reg_{j}\right)+\frac{\sum_{\left(x,y\right)\in box_{i}}Act^{C}\left(x,y\right)}{N_{\left(x,y\right)\in box_{i}}}$$ (3)

Here $N_{(x,y)\in box_{i}}$ denotes the number of activation points in *box*_*i*_, and ${\text{Act}}^{\text{C}}$ represents the CAM for extracting *box*_*i*_ , where *C* is its channel category.

However, the close proximity between the instrument and the target it acts upon can cause confusion in matching when using only the Intersection over Union (IOU) as the overlap metric for $box\in Box_{r}$ and $reg\in Reg_{s}$. To tackle this, we use a ResNet18-based network to classify $reg\in Reg_{s}$ into *Instrument* and *Tissue*. This lightweight model is trained on approximately 300 SAM candidate regions labled as *Instrument* and *Tissue*. Our definition of *Overlap* is as following Eq 4:

Overlap(box,reg)=[I(box,reg)+T(box,reg)]*[CIoU(box,reg)+K] (4)

$$I\left(box,reg\right)=\left\{ \begin{array}{c} 1\;\text{if}\;\left\{c_{box}\in Instrument\right\}\text{and}\left\{p_{reg}=Instrument\right\}\\ 0\;\text{otherwise} \end{array}\right.$$

$$T\left(box,reg\right)=\left\{ \begin{array}{c} 1\;\text{if}\;\left\{c_{box}\in Target\right\}\text{and}\left\{p_{reg}=Tissue\right\}\\ 0\;\text{otherwise} \end{array}\right.$$ (5)

*I*(*box*,*reg*) and *T*(*box*,*reg*) are defined as Eq 5, where *c*_*box*_ is the category of *box*, *p*_*reg*_ is the classification prediction for *reg* and *K* is a positive constant that keeps CIoU(box,reg)  +  *K* always positive. Through this approach, the risk of mismatching could be reduced. For example, a *reg* categorized as *Instrument* and a *box* of *Target* will not be matched together, since in this case their *Overlap* would be 0. *CIoU*(*b*,*r*), referring to the simplified *CIoU* [28], is defined as follows:

$$CIoU\left(box,reg\right)=IoU\left(box,reg\right)-\frac{\rho^{2}\left(box,reg\right)}{c^{2}}$$ (6)

Where ρ(box,reg) represents the distance between the central points of *box* and *reg*, and *c* denotes the diagonal length of the minimum enclosing rectangle for both.

Ultimately, We derive a collection of matched pairs $M=\{(m,n)\;|\;box_{m}\in Box_{r},reg_{n}\in Reg_{s}\}$. For each matched *reg*_*n*_, its bounding box is extracted as the localization result, with its class inherited from the category of *box*_*m*_ ’s source CAM.

## Results and discussion

### Datasets and evaluation metrics

#### Training dataset.

CholecT50 [18] is a publicly available dataset of endoscopic videos of laparoscopic cholecystectomy surgery, introduced to support research on fine-grained actions in laparoscopic surgery. The dataset consists of 50 videos, each annotated by two surgeons with triplet information in the form of ⟨instrument, verb, target⟩ for each frame. It contains binary presence labels for 6 instruments, 10 verbs, 15 targets, and 100 triplet categories. Our RDV-AGC is trained on 40 videos and validated on 5 videos from the CholecT50 dataset. The video IDs are detailed in Table 1.

**Table 1:** The details of dataset splits.

	Dataset Splits (CholecT50 Video IDs)
**Training**	**01, 02, 04, 05, 06, 08, 10, 12, 14, 15, 18, 22, 23, 25, 26, 27, 31, 32, 35, 36, 40, 42, 43, 47, 49, 50, 51, 52, 56, 57, 60, 62, 66, 78, 79, 80, 92, 96, 103, 110**
**Validation**	**13, 29, 48, 65, 111**
**Evaluation**	**68, 70, 73, 74, 75**

#### Evaluation dataset.

To evaluate localization for surgical components, we construct a dataset based on the validation set from the CholecTriplet 2022 Challenge [8] used for the instrument localization task. This dataset involves 5 video clips from CholecT50 [18] with the labels of triplet binary presence and instruments’ bounding boxes. The specific video IDs are shown in Table 1. The uniqueness of our evaluation dataset lies in: (1) We not only draw bounding boxes for the 6 types of instruments in the video clips but also annotate the spatial bounds for 12 types of targets, thus addressing the lack of target localization annotations in [8,18]. (2) Instead of only considering the effector of the instrument, we annotate for the whole instrument to achieve complete localization.

The spatial annotations in the form of bounding boxes are drawn using the LabelImg [29] annotation tool by two professionals, who have been trained by a team of surgeons. These spatial annotations are merged with the existing triplet binary presence labels and stored in JSON format. Our evaluation dataset contains bounding box annotations for surgical components from a total of 902 video frames and will be publicly available with this paper. Table 2 presents the bounding box instances counts for surgical components in our evaluation dataset.

**Table 2:** Statistic of the bounding box instances in evaluation set.

Instrument	Count	Target	Count
grasper	318	gallbladder	259
irrigator	243	fluid	110
hook	53	cystic-plate	87
bipolar	232	cystic-duct	259
clipper	99	cystic-pedicle	42
scissor	97	cystic-artery	114
		specimen-bag	55
		liver	96
		omentum	33
		admominal wall cavity	55
		gut	3
		blood vessal	23

#### Evaluation metrics.

The method performance is assessed using the average precision (AP) and average recall (AR). These metrics are computed based on precision (*p*) and recall (*r*) scores as follows:

$$p=\frac{TP}{TP+FP},\;r=\frac{TP}{TP+FN}$$ (7)

Where TP, FP, and FN refer to true positives, false positives, and false negatives respectively. In the instrument and target detection task, a detection is considered TP if it satisfies both conditions: the predicted class matches the ground truth class, and the IoU between the predicted bounding box and the ground truth exceeds a certain threshold. If either condition is violated, the detection is assigned as FP, while missing detections corresponding to the ground truth are marked as FN. For each IoU threshold, *p* and *r* are computed, and *p*-*r* curves and *r*-*IoU* curves are plotted. AP and AR are obtained by calculating the area under the *p*-*r* curve and twice the area under the *r*-*IoU* curve, respectively:

$$AP=\int_{0}^{1}p(r)dr,\;AR=2\int_{0.5}^{1}r(iou)d(iou)$$ (8)

In our evaluation, we compute the final performance metric by averaging the AP and AR values across all *K* classes.

$$mAP=\frac{1}{K}\sum_{i=1}^{K}AP_{i},\;mAR=\frac{1}{K}\sum_{i=1}^{K}AR_{i}$$ (9)

### Implementation details

First, we train the recognition network RDV-AGC on our training dataset. Then, we use the RDV-AGC to extract positive activation for each input video frame. The areas of positive activation will be matched with the candidate regions generated by SAM finally. During the stage of training RDV-AGC, we initially unified the spatial dimensions of input frames by resizing them to 256×448. We also employed random horizontal/vertical flips and random brightness/contrast shift as data augmentation. The resolution of the output CAMs from both instrument and target branches in RDV-AGC are set to 8×14. We initialize the Feature Extractor module and WSL module with parameters from RDV which was pre-trained on our training set for 100 epochs. All modules in RDV-AGC are trained using stochastic gradient descent with momentum (μ=0.95) as the optimizer. We implement a sequential combination of learning rate schedule strategy, which includes a linear warm-up scheduler before milestones, followed by an exponential decay (γ=0.99) after milestones. Our model is trained for 50 epochs with a batch size of 32. For the generation of SAM candidate regions, we initialize SAM with parameters pre-trained on the SA-1B dataset [10] and set to ’vit_h’ scale.

Our network is implemented in PyTorch and performed on NVIDIA RTX A6000. Full training for RDV-AGC takes approximately 65-80 hours on a single RTX A6000. The total storage space consumption for RDV-AGC, SAM, input data, output weights, and SAM candidate masks is about 15GB.

### Comparison against similar works

**Fig 4:**
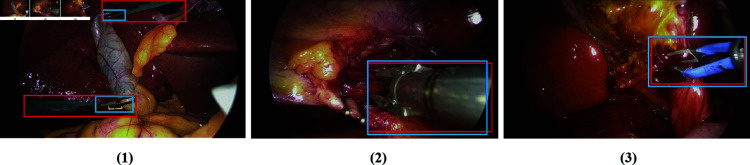
The examples of annotation: In (1), the bounding boxes annotated in the two datasets differ significantly, whereas in (2) and (3), the annotations from both datasets largely overlap. The blue bounding boxes are from dataset in [8], and the red ones are from ours.

We compare the proposed method with other works focused on instrument detection. These include both supervised methods [7,9] that rely on spatially annotated training data and weakly supervised methods [8] that do not require such annotations. The results of these methods on our evaluation dataset (902 frames) are presented in Table 3. However, it is important to note that supervised methods, such as Faster R-CNN [7] and MCIT+IG [9], use large-scale training datasets where only the effectors (tips) of the instruments were spatially annotated, like the m2cai16-tool-locations dataset [7] used in [7,9]. This causes the trained models to focus more on locating the effectors of the instruments. In contrast, our proposed method and evaluation dataset are both designed for locating the complete instrument regions in the frame. The inconsistency between these two types of annotations is illustrated in Fig 4. Given this, directly comparing detection performance using the entire evaluation dataset may not be entirely fair.

**Table 3:** Comparison with similar methods for instrument detection on evaluation (eval) set (902 frames) and subset (279 frames) (mAP@0.5 in %).

Method	Annotated	Eval Set		Eval Subset	
	Frames	$mAP_{I}$	$mAR_{I}$	$mAP_{I}$	$mAR_{I}$
Faster R-CNN [7]	~ 23000	32.5	36.3	50.3	54.1
MCIT+IG [9]	4214	21.9	26.8	34.6	39.2
MCIT+IG [9]	15316	35.0	38.3	53.8	58.9
MCIT+IG [9]	24536	40.7	43.2	61.5	67.6
DualMFFNet [8]	0	4.5	6.9	5.0	7.6
MTTT [8]	0	11.6	18.0	12.3	21.9
Ours	0	51.1	48.5	51.7	50.3

To ensure a more reasonable comparison, we create an additional evaluation subset containing 279 frames, where the instrument annotations are suitable for evaluation across all methods. Specifically, we analyze our evaluation dataset against the original dataset from the CholecTriplet 2022 Challenge [8], which also only annotate the effector parts of the instruments. We select frames where the bounding box annotations for the instruments are highly consistent (average IoU > 0.85) between the two datasets. As shown in Fig 4 (2) and (3), when only the effector part of the instrument is visible, the localization boxes from both annotation types overlap significantly. We perform additional evaluations on this selected subset for all similar methods, and the results are also presented in Table 3. Notably, as other similar methods have yet to explore target localization, we do not present the results of target detection in this section.

According to Table 3, Our method shows outstanding performance on instrument detection, notably exceeding other weakly supervised approaches: outperforming DualMFFNet [8] by +46.6% mean Average Precision (mAP) on evaluation set (902 frames) and +46.7% on subset (279 frames) , and exceeding MTTT [8] by +39.5% mAP on evaluation set and +39.4% mAP on subset. Compared to supervised methods that require spatial annotations, our method surpasses MCIT+IG [9], trained on approximately 4,000 spatial annotated frames, by +29.2% mAP on evaluation set and + 17.1% mAP on subset. It is comparable in accuracy to both MCIT+IG [9] trained on about 15,000 annotated frames and Faster R-CNN [7] trained on approximately 23,000 frames on evaluation subset. Although it underperforms MCIT+IG [9] trained with around 25,000 frames by –9.8% mAP on evaluation subset, our method significantly reduces the need for extensive spatial annotations.

### Qualitative experiment results

**Fig 5:**
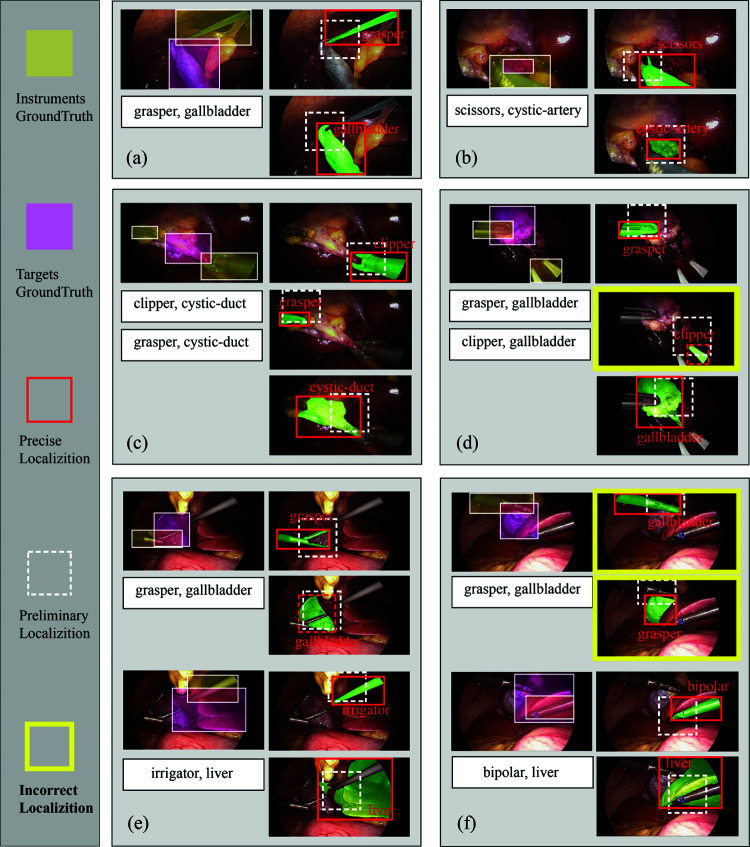
Qualitative experiment results: Ground truth and our method’s predictions on 6 frames (a)-(f) from the validation set. Frame (a)-(b) contain only one pair of surgical components. Frame (c)-(d) each has two pairs of surgical components with the same target for both pairs. Frame (e)-(f) also contain two pairs of surgical components, but with different targets for each pair. Images highlighted with yellow borders show incorrect detection results.

The localization of surgical components on evaluation dataset, depicted by bounding boxes overlaid on the frames, is shown in Fig 5. The proposed method accurately locates instruments and targets in most cases. It occasionally fails in several situations, such as only partially detecting an instrument as shown in Fig 5 (d), or incorrectly identifying targets as instruments in Fig 5 (f). The latter error occurs when the model fails to distinguish candidate regions of instruments and targets during the matching stage.

### Ablation studies

#### Ablation study on crucial modules.

To demonstrate the superiority of our method in localizing instruments and targets, we begin with an ablation study on the crucial modules in our model. The presence or absence of the AGC and CRM modules corresponds to the following four methods:

1. RDV-det: This is a detection version of RDV [4] model, which learns the location of the 6 distinct instruments and 15 distinct targets in the CholecT50 dataset from the last 6-channel convolutional layer of the WSL and the last 15-channel convolutional layer of the target branch in CAGAM. Specifically, it extracts bounding boxes for every positive activation in the CAMs channel-wisely and applies non-maximum suppression (NMS) to remove redundant objects.

2. RDV-AGC-det: It integrates our AGC module into RDV, following the description in ***Target Class Activation Correction***. Then the RDV-AGC model is used to locate surgical components in the same manner as RDV-det.

3. RDV + CRM: It first generates SAM candidate regions following the pipeline in ***SAM Candidate Regions Generation***, then preliminarily locates surgical components by extracting the positive activation of the CAMs from RDV. Finally it uses CRM to match candidate regions and preliminarily locations as described in ***Precise Localization***.

4. RDV-AGC + CRM: Our proposed method combines the RDV-AGC recognition network with the CRM mechanism.

For fair comparison, we select the best weights after 150 epochs of training for RDV, and the best weights after 50 epochs for RDV-AGC. Both RDV and RDV-AGC are trained on our training set. The matched SAM candidate regions are extracted in the form of bounding boxes as the final localization results.

Table 4 shows the quantitative results of the four above-mentioned methods with Average Precision and Average Recall for instrument and target detection. Drawing on SAM’s powerful zero-shot learning capability, our method provides more accurate localization than CAM-based method, improving the performance of instruments detection by +48.86% mAP, which is 23.5 times increase in the RDV-AGC-det method. Furthermore, by correcting the attention guide for targets, AGC module improves the performance of our method on target detection by +16.51% mAP, which is 4.04 times increase in the RDV-AGC + CRM method.

**Table 4:** Ablation study results on crucial modules (mAP@0.5 in %).

Method	Instrument Detection		Target Detection	
	$mAP_{I}$	$mAR_{I}$	$mAP_{T}$	$mAR_{T}$
RDV-det	2.04	5.06	0.16	0.58
RDV-AGC-det	2.56	5.20	0.42	1.02
RDV + CRM	46.84	48.62	5.02	10.80
RDV-AGC + CRM (Ours)	51.13	48.52	21.47	23.31

**Fig 6:**
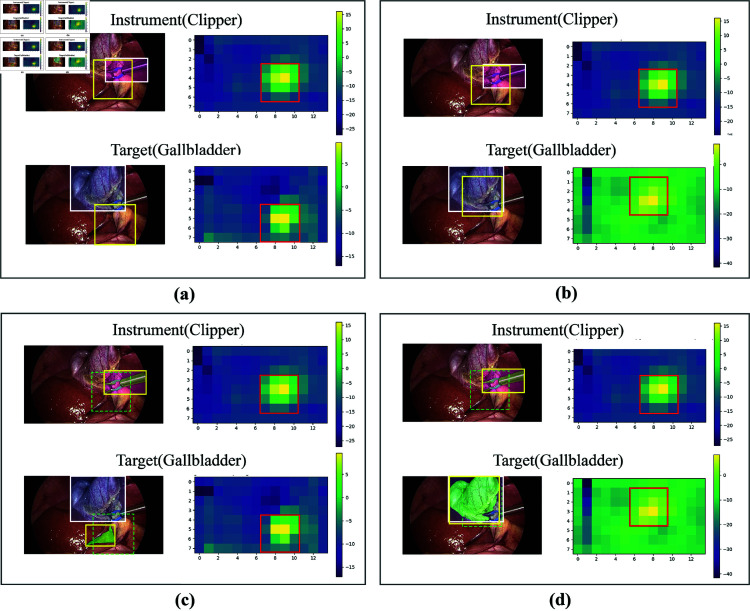
The comparison of our method with baselines on the localization results. Each subfigure represents: (a) RDV-det; (b) RDV-AGC-det; (c) RDV + CRM; (d) Ours. Different colored boxes represent: white = ground truth, yellow = final localization result, green (dashed line) = preliminary localization box, red = bound of the positive activation region in CAM.

Some predicted results in Fig 6 intuitively show that the AGC module brings the positive activation of targets closer to their true positions. As shown in Fig 6 (a) and Fig 6 (b), after the introduction of the AGC module, the positive activation in resulting CAM for target (gallbladder) is precisely adjusted to the upper left side of the instrument (clipper). Moreover, a comparison between Fig 6 (a) and Fig 6 (c) also reveals that the CRM mechanism significantly enhances the accuracy of localization by correctly matching the SAM candidate regions with the CAM-based localization boxes.

**Fig 7:**
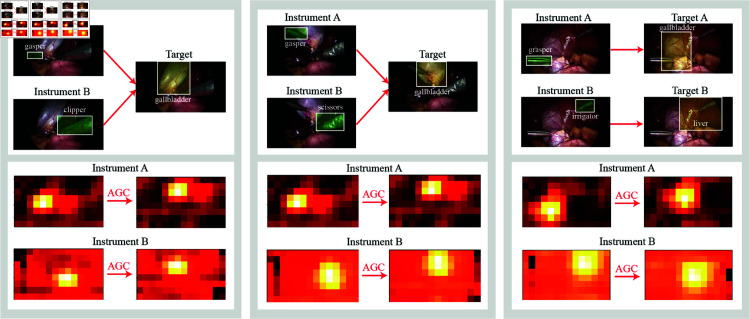
Three instances: Through AGC module, the positive activation regions of multiple instruments are brought closer to the actual positions of their corresponding targets.

Additionally, Fig 7 provides an intuitive illustration of the AGC module’s operation and its effectiveness in offering a more accurate guide for target detection. As shown in Fig 7, the AGC module brings the positive activation regions of multiple instruments closer to the actual positions of their corresponding targets.

#### Selection of important hyper-parameters.

This section analyses the absolute values of the crucial parameters *K*_*h*_ and $K_{v}$ in the AGC module. According to the description for AGC module in ***Target Class Activation Correction***, the effectiveness of the correction on the target’s position attention guide improves as the translation distance of the instrument’s CAM more closely approximates the actual distance between instrument and target projected on the CAM. Therefore, we initially perform a statistical analysis of the spatial distances between the instrument and the target within the same triplet in laparoscopic cholecystectomy video frames. This analysis is aimed at determining the typical range and central tendency of these distances in such surgical scenes, which will help refine the specified range of interest for |*K*_*h*_| and $\left|K_{v}\right|$.

**Fig 8:**
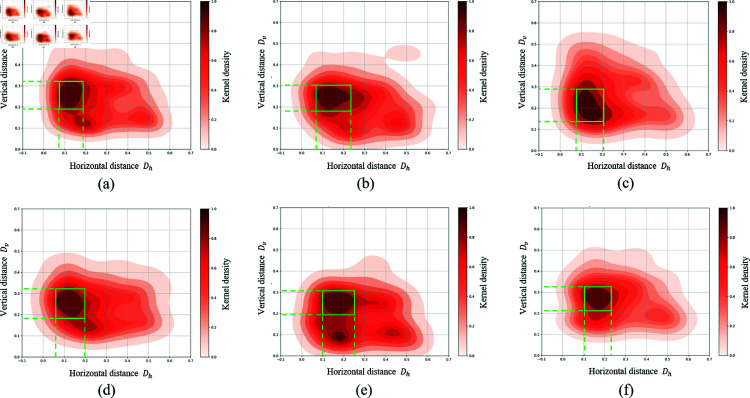
The Kernel density estimation (KDE) heat maps of *D*_*h*_ and $D_{v}$: (a) Total data; (b)-(f): The $1^{st}\sim 5^{th}$ fold. The green bounding boxes highlight the highest density areas.

Our analysis is conducted on evaluation data annotated with spatial locations. We calculate the horizontal distance *D*_*h*_ and vertical distance $D_{v}$ between the central points of the bounding boxes for instruments and targets within each triplet, scaled by the dimensions of the video frame. We randomly divide the evaluation data into five folds. Fig 8 shows the Kernel Density Estimation (KDE) [30] heat maps of *D*_*h*_ and $D_{v}$ for both the total data and each individual fold, illustrating their distribution characteristics. Fig 8 (a)-(f) reveal that the density distributions of *D*_*h*_ and $D_{v}$ are highly consistent across all folds, with the highest density areas primarily concentrated within $D_{h}\in (0.1,0.2)$ and $D_{v}\in (0.1,0.3)$.

**Fig 9:**
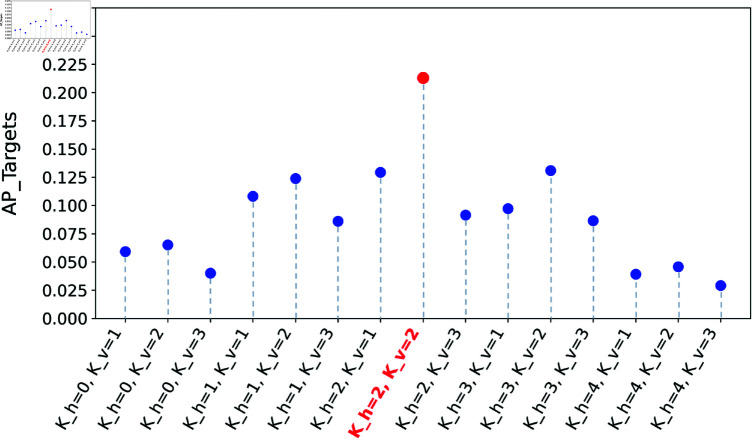
The target detection performance across various combinations of |*K*_*h*_| and $\left|K_{v}\right|$ within the specified interest ranges.

Considering the highest distribution density ranges of *D*_*h*_ ,$D_{v}$ and the dimension of the CAMs in the RDV-AGC, we select $K_{h}\in [0,4]$ and $K_{v}\in [1,3]$ as the ranges of interest for |*K*_*h*_| and $\left|K_{v}\right|$. The model performance across various combinations of |*K*_*h*_| and $\left|K_{v}\right|$ within the specified interest ranges is showed in Fig 9. The model achieves highest accuracy for target detection when both |*K*_*h*_| and $\left|K_{v}\right|$ are set to 2, with performance diminishing as the values deviate from this optimal setting. Therefore, both |*K*_*h*_| and $\left|K_{v}\right|$ are selected to 2 for AGC module to achieve best performance in laparoscopic cholecystectomy scenes. Notably, the optimal values of *K*_*h*_ and $K_{v}$ correspond to the observed trends in distances between instruments and targets in the visual field of laparoscopic cholecystectomy.

#### Ablation study on CAM resolution.

We apply CAMs with different resolutions (8×14, 16×28, 32×56) for *Target Class Activation Correction* and *Preliminary Localization*. Similarly, |*K*_*h*_| and $\left|K_{v}\right|$ are set to 2×2, 4×4, and 8×8, respectively. The models with these varying CAM resolutions are evaluated on the evaluation dataset, and the corresponding detection performance is shown in Table 5.

**Table 5:** Ablation study results on CAM resolution (mAP@0.5 in %).

Resolution	Instrument Detection		Target Detection	
	$mAP_{I}$	$mAR_{I}$	$mAP_{T}$	$mAR_{T}$
8×14	51.13	48.52	21.47	23.31
16×28	51.25	49.23	20.85	22.96
32×56	51.04	49.16	20.38	23.22

As presented in Table 5, increasing the resolution of the output CAM do not lead to significant improvements in the detection performance of both instruments and targets. Notably, the AP and AR for target detection even decline slightly, possibly due to the increased resolution introducing redundant information when localizing the positive activation areas. More importantly, higher-resolution CAMs require more computational resources, resulting in lower training efficiency. Therefore, we set the output CAM resolution to 8×14 for all the experiments conducted in this study.

## Conclusion

In this work, we propose an innovative method for detection of critical surgical components. For CAM-based preliminary localization, we introduce RDV-AGC, which incorporates an attention guide correction module to achieve more accurate localization for targets. To precise localization, we propose CRM mechanism. Through accurate matching of candidate regions generated by SAM and CAM-based preliminary localization, we facilitate the precise detection of surgical components with no need for spatial annotations. To evaluate our method, we also annotate spatial bounds for instruments and targets from 902 frames. Both quantitative and qualitative results validate our superiority. While these initial results are encouraging, some limitations remain as follows:

1. Although SAM can generate relatively accurate segmentation masks for surgical components with appropriate prompts, issues such as unstable segmentation edges persist. We have not yet achieved pixel-level segmentation of surgical components.
2. The detection for targets has considerable room for improvement due to their ambiguous boundaries and low discriminative features.
3. The translation distances |*K*_*h*_| and $\left|K_{v}\right|$ in the AGC module require re-evaluation and adjustment for different domains, which impacts the model’s adaptability.

Based on this work, future work will consider the pixel-level segmentation of surgical components. Moreover, we will study the localization and segmentation of richer anatomy in surgical scenes in the future.

## Supporting information

s1 Appendix The detail of multi-task loss *L*_*comp*_ (PDF)

S1 Table The value of mentioned parameters. (PDF)
