# Supplementary material for: A weakly supervised method for surgical scene components detection with visual foundation model
Source: PLoS One. 2025 May 27;20(5):e0322751. doi: 10.1371/journal.pone.0322751 (PMC12111666; doi:10.1371/journal.pone.0322751)
Supplement: s1 Appendix — (PDF) [file pone.0322751.s001.pdf]

S1 Appendix

The detail of multi-task loss L_{comp}

L_{comp} denotes the multi-label loss used for classifying each triplet component. It is composed of the weighted sigmoid cross-entropy losses for each component: L_I for the instrument, L_V for the verb, and L_T for the target. The weighted cross-entropy with logits is as follows:

$$L = \sum_{\mathrm{c}=1}^{C} \frac{-1}{N} \left(W_c y_c \log \left(\sigma \left(\hat{y}_c \right) \right) + \left(1 - y_c \right) \log \left(1 - \sigma \left(\hat{y}_c \right) \right) \right) \tag{S1}$$

where y_c and $\hat{y}_c$ are respectively the ground truth and predicted labels for class c. σ represents the sigmoid function, and W_c is a weight designed for class balancing. The multi-task learning of these three component classify tasks is following the uncertainly loss procedure that uses learnable parameters w_I, w_V, w_T to automatically balance the tasks training. The equation for L_{comp} is given below:

$$L_{comp} = \frac{1}{3} \left(\frac{1}{e^{W_I}} L_I + \frac{1}{e^{W_V}} L_V + \frac{1}{e^{W_T}} L_T + W_I + W_V + W_T \right) \tag{S2}$$
