# Supplementary material for: A weakly supervised method for surgical scene components detection with visual foundation model
Source: PLoS One. 2025 May 27;20(5):e0322751. doi: 10.1371/journal.pone.0322751 (PMC12111666; doi:10.1371/journal.pone.0322751)
Supplement: S1 Table — (PDF) [file pone.0322751.s002.pdf]

# S1 Table

## The value of mentioned parameters

Table S1: The value of mentioned paremeters.

| Section | Parameter | Name | Value |
|---|---|---|---|
| Sect 3.1 | Number of foreground points to prompt SAM | $N \times N$ | $32 \times 32$ |
| Sect 3.1 | Prediction IoU threshold | $T_{iou}$ | 0.8 |
| Sect 3.1 | NMS threshold for SAM regions filtering | $T_r$ | 0.7 |
| Sect 3.2 | The regularization weight decay for the $L_2$ normalization loss | $\lambda$ | $1e^{-5}$ |
| Sect 3.3 | NMS threshold for boxes filtering | $T_b$ | 0.7 |
| Sect 3.4 | The positive constant to keep $CIOU(box + reg) + K$ positive | $K$ | 1 |
